# Participatory research with co-researchers with lived experience of psychosis high risk states

**DOI:** 10.3389/fpsyt.2025.1530093

**Published:** 2025-06-02

**Authors:** Barbara Hinterbuchinger, Raphaela E. Kaisler, Josef S. Baumgartner, Fabian Friedrich, Zsuzsa Litvan, Melanie Trimmel, Karin Hlavacek, Alina Ramya Popa, Nilufar Mossaheb, The VOICE Consortium

**Affiliations:** ^1^ Clinical Division of Social Psychiatry, Department of Psychiatry and Psychotherapy, Medical University of Vienna, Vienna, Austria; ^2^ Comprehensive Center for Clinical Neurosciences and Mental Health, Medical University of Vienna, Vienna, Austria; ^3^ Department of Psychotherapy, Bertha von Suttner Private University St. Pölten GmbH, St.Pölten, Austria; ^4^ ESRA, Psychosocial Centre, Vienna, Austria

**Keywords:** participatory research, psychosis high risk, ultra-high risk for psychosis, service user involvement, experts by experience

## Abstract

**Background:**

Although in psychiatric research prevention and participation are both considered increasingly important, there are few participatory research projects with individuals with psychosis high risk states (ultra-high risk for psychosis; UHR). The aim of this project was to reflect on UHR terminology, diagnostic and treatment guidelines and to identify and implement unmet needs together with people at UHR.

**Methods:**

This project was designed co-creatively from the conceptual phase to the execution. The project team consisted of an equal number of mental health clinicians and co-researchers with lived UHR experience. Rules for collaboration were co-creatively developed within the group. Within 4 project workshops, project objectives and unmet needs were identified and prioritized. After setting up an action plan, project plans were implemented within the research group.

**Results:**

Unmet needs of co-researchers with lived UHR-experience included free access to information on psychosis high risk states, opportunities for personal exchange, and the creation of more public awareness and knowledge about UHR. Within the participatory research process, consensus on collaboration and objectives was achieved and heterogeneous perceptions towards the UHR concept and terminology were discussed.

**Consensus:**

The necessity of an adequate terminology for psychiatric conditions was deemed crucial by both medical professionals and co-researchers with lived UHR experience for facilitating a better understanding between psychiatrists and those affected. Heterogeneity of perception illustrates the necessity of addressing individual needs and utilising diverse terminology and explanatory models within mental health.

## 1 Background

People have the right to participate in decisions that affect their lives (1). Public and patient involvement and engagement (PPIE), which overlaps in content and definition with ‘participatory research’ and ‘service user involvement’, has the potential to democratize research processes and introduce a shift in power and ownership towards public members (2–4). Levels of participatory research involvement can range from consultation (researchers seeking feedback from people with lived experience) to collaboration (co-production of research) and user-lead research (people with lived experience are in control of research) (5, 6). Over the past few decades, participation in psychiatry has marked a shift towards involving people with lived experience of mental illness (PWLE) as active participants in mental health research, recognizing their personal experiences as expertise (4). There is growing recognition of the importance of PPIE in psychiatry research, i.e., the National Institute for Health and Care Research (NIHR) INVOLVE framework defining best practices for PPIE in health research with public involvement in its principles from its beginning in 2006 (7). Furthermore, PPIE is a required component of many funding grants. The inclusion of co-researchers with lived experience in psychiatric research is supported by both epistemic and ethical justifications. Some argue that those directly impacted should be able to contribute to psychiatric research in the interest of “nothing about us without us”, while others prioritize the value of gaining new and changing existing knowledge in research (8, 9). Indeed, the Lancet Commission on Ending Stigma and Discrimination in Public Health found that the involvement of PWLE as co-producers is the most important factor in stigma reduction interventions (10). Challenges associated with PPIE include a lack of knowledge that can result in a feeling of being unable to contribute in a meaningful way (11), power imbalances between researchers and service users (12) as was well as tokenistic involvement (13) and financial and structural barriers, i.e., lack of funding or institutional support (14, 15).

The prevention of severe psychiatric disorders, such as psychotic disorders, is considered highly relevant (16, 17) with respect to the negative effects of these disorders on disability-adjusted life years (DALY) (18), decreased life expectancy (19) and impairments concerning quality of life (20). Research on indicated prevention of psychosis began about 25 years ago (21). Indicated prevention in psychosis high risk states was found to reduce existing symptoms, delay or potentially prevent transition towards manifest psychosis, reduce health care utilization, and shorten the duration of untreated psychosis (17, 22, 23). The operationalized criteria for identifying individuals at “ultra-high risk for psychosis” (UHR) or “clinically high risk for psychosis” (CHR) were developed to identify an increased risk for the development of manifest psychosis in help-seeking individuals with distress (24, 25), i.e., the basis for preventative research. UHR criteria consist of one genetic and deterioration criterion (GRDS) and two criteria that include distressing experiences below the threshold of manifest psychotic symptoms either with respect to time or with respect to frequency and intensity: “attenuated psychotic symptoms” (APS) and “brief limited psychotic symptoms” (BLIPS) (24). Initial studies on transition rates in UHR individuals showed a rate of 30% of first episode psychosis onsets within one year (26). However, in recent years, transition rates have decreased to about 25% at three years (27), bringing about a critical discussion: Some have questioned the validity of the concept itself and raised concerns about possible stigmatization with associated terms such as “prodromal”, “risk” and “early detection” (28). Other have argued for the validity and clinical utility of the UHR/CHR construct, expanding it to a transdiagnostic at-risk mental state (29). Indeed, risk recognition is a key task in preventative medicine and implies early intervention measures of risk and symptom. In light of the mentioned discussion, a collaboration with individuals with lived UHR-experience is highly necessary to discuss and reflect on UHR terminology and therapeutic and diagnostic interventions, as well as address unmet needs and personal insights of those with lived experience. A recent review, co-authored by experts with lived experience, describes the importance of acknowledging PWLE’s perspectives and experiences across the clinical stages of psychosis (30). While participatory research with people with psychotic disorders at various levels of involvement and collaboration (31–33) does exist, there are few participatory research studies (34, 35) with people at psychosis high risk states as co-researchers.

In the current paper, we present the participatory research process of the VOICE project and its outcomes. The VOICE project is a co-creation research project including mental health professionals and co-researchers with lived experience of UHR. Within this participatory project, we aimed to identify unmet needs of those with lived UHR experience, to implement project objectives based on unmet needs and to reflect on the psychosis high risk concept, UHR terminology and diagnostic and treatment options. A particular strength of the VOICE project was its open and fluid process. Consequently, the jointly developed project goals and outcomes were partly subject to change during the research process.

## 2 Materials and methods

### 2.1 Recruitment of co-researchers with lived experience of UHR

The project design and application were followed through by two psychiatrists and a co-researcher with lived experience of UHR. All further decisions in the recruitment process were discussed between these three researchers. Co-researchers with lived experience of “ultra-high risk for psychosis” (UHR) were recruited at the Early Psychosis outpatient clinic, specialized in the early detection and treatment of psychosis risk states, at the Clinical Division of Social Psychiatry, Department of Psychiatry and Psychotherapy, Medical University of Vienna. Recruitment was conducted by two experienced psychiatrists from the early intervention UHR outpatient clinic. Individuals with a lifetime history of UHR that attended the early intervention UHR outpatient clinic were provided information on the possibility of participation in this project. The decision to participate or decline did not affect the participants’ subsequent treatment. Participants were free to withdraw from the project at any time. The recruitment objective was to achieve a balanced ratio of male and female participants. We further aimed for diversity concerning the background of participants. To be included as project co-researchers, individuals with a lifetime history of UHR had to be free of recent suicidal behavior or suicidality, without a lifetime manifest psychotic episode and WITH availability to participate in the project. Participants had to be at least 18 years old.

### 2.2 Project governance: core team and study advisory group

The Core Team of the project, also referred to as the project steering committee, consisted of two co-researchers with lived experience of UHR and two psychiatrists with experience in research and clinical work on early psychosis and UHR. One of the co-researchers with lived UHR experience was involved in co-writing the project proposal and grant application during the project’s ideation phase. The second co-researcher of the Core team was recruited after the project was approved for funding by the Ludwig Boltzmann Gesellschaft (LBG) (https://lbg.ac.at). The Core team was responsible for establishing the framework for collaboration within the project, e.g., developing a safety plan in case of psychiatric deterioration within the project period, agreeing on rules for confidentiality and communication within and between the workshops, as well as responsibilities and decision-making processes within the core team.

In addition to the Core team, the Study Advisory Group was formed with a total of eight people, consisting of four people with lived experience of UHR and four psychiatrists or psychiatric residents with clinical experience in early psychosis and psychosis risk states. The framework and conditions for safety, communication, confidentiality, anonymity and informed consent were further discussed with the Study Advisory Group until full consensus was reached among all project participants during the first workshop.

### 2.3 The co-creation process and data collection

The co-creation process consisted of four workshops: One to establish the framework for the project and to create a collaborative working atmosphere, and three thematic workshops to explore the concept of UHR, terminology, diagnostic and treatment options. Co-researchers with lived experience received payment (honoraria) or remuneration (e.g., vouchers) to value their time, skills and knowledge and to reduce potential power imbalances between professionals and co-researchers with lived experience. If participants could not join the workshops personally, the opportunity for online participation was given. The four full-day workshops were held in a pleasant venue for meetings in a hotel in Vienna between June and November 2022. A facilitator experienced in implementing PPIE processes moderated and documented the workshops as agreed upon with the Core team in an iterative manner. All workshops included the following elements: a) provision of information (capacity building) on specific topics (20 minutes), b) one-on-one or small group interactions between clinicians/researchers and co-researchers to share experiences and foster mutual learning, c) co-creation of content on specific topics in small groups using methods such as the Open Space Method, World Café (group discussion) and Gallery Walk, d) and group discussions to reach consensus on specific topics.

The first co-creation workshop set up rules for collaboration concerning confidentiality, anonymisation, informed consent, decision-making, a safety plan, communication structures and roles and tasks of the team. The project’s vision was defined, exploring values and benefits for different stakeholder groups (e.g., clinicians, patients, family members, the general public), project aims and objectives. Also, possibilities to evaluate the workshops considering PPIE elements were discussed and agreed upon. In the second workshop project objectives and aims were further prioritized with a scoring-based system. All participants could distribute points to make an individual assessment of the topics of interest. Based on the evaluation of the participants, a ranking of the topics of interest was created by counting the points awarded. Assessment of UHR-criteria in help-seeking individuals were discussed within small groups consisting of clinicians and co-researchers. Individuals shared insights and highlights of small-group discussions with the whole group and framed general considerations for UHR assessment. The third workshop explored terminology of psychosis risk-related terms and definitions. Participants reflected own experiences and meaning of familiar terms and arranged their insights on a grid in 4 categories (helpful and tolerable/stressful but endurable/not endurable and burdensome/not endurable but helpful). A consensus was reached regarding specific terms and discussions were led on those that were perceived with higher discrepancy. The fourth and last workshop addressed existing intervention options and recommendations for people with UHR-experience. Again, both clinical researchers and researchers with lived experience discussed their individual experiences in small groups and shared their discussion points and insights with the whole group. The second part of the workshop was dedicated to the collaborative analysis of previously written reflections. Contents of the texts were clustered into categories and subcategories creating a coding tree for further comparative analysis. Lastly, the group reflected on the co-creative experience and discussed take-home messages for researchers/clinicians and co-researchers. Based on the ongoing dialogue, discussion and exchange within the workshops, unmet needs were identified. Strategies to address these needs and an action plan for implementation were developed taking into consideration participants’ individual skills and preferences.

For a comprehensive description see “VOICE - PPIE with people at ultra-high risk for psychosis - A “How to” Guide for researchers” (36).

#### 2.3.1 Participants

A total of twelve participants, including the Core Team and the Study Advisory Group (age M = 31.45 years, SD = 9.07; 67% female), thereof six psychiatrists or psychiatric residents experienced in clinical treatment of individuals with psychotic disorders or psychosis high risk states and six co-researchers with lived experience of UHR, with diverse backgrounds participated in the workshops. Half of the participants were employed at the university, three in the public sector, two had no and one another employment. Six participants reported having expertise in the area of health and medicine (i.e., the six psychiatric professionals) and two in social sciences and humanities. None of the twelve participants had prior experience with PPIE in research. The group remained consistent throughout the project process. Written informed consent was obtained from all research participants.

#### 2.3.2 Evaluation of workshops

At the end of each workshop, participants completed a short self-report questionnaire adopted from the LBG participation check (https://ois.lbg.ac.at/ois-resources/tools/) assessing the collaboration between researchers/clinicians and co-researchers on a closed-ended questions with a 5-point Likert scale (1 ‘not at all satisfied’ – 5 ‘very satisfied’ or 1 ‘does not apply at all’ – 5 ‘strongly applies). In addition, a 5-point semantic differential was used to assess the atmosphere in the workshops, e.g., boring - exciting. The dimensions of the questionnaire included demographic data, impressions of the atmosphere in the workshop, satisfaction with the workshop, takeaways from the workshop, involvement in the workshop, the overall satisfaction, and aspects of co-creation in the workshop (for further information see 36; Chapter 2.3.2.3.). Descriptive statistical analysis of the data was performed with Microsoft Excel calculating average means (*M*) and standard deviations (*SD*) of each item.

## 3 Results

Results can be divided into those concerning (i) description and evaluation of the co-production process (ii) project outcomes (unmet needs/reflection on terminology/diagnostic and treatment options).

### 3.1 Co-production project process

#### 3.1.1 Consensus on project collaboration

Consensus was reached on behavioural rules for teamwork, communication and project collaboration, with confidentiality, anonymisation of data and the use of consent forms all considered relevant by the participants. The self-determination of what biographical and personal information was revealed in the workshops was upheld by all participants, as was the non-transfer of information given in the workshops to third parties. It was agreed that no personal details of medical treatment should be discussed in the workshops, nor should they primarily serve the purpose of self-experience or supervision. Additionally, a set of non-negotiable principles for collaboration were established including respect of boundaries, of work settings and general trustworthiness. Furthermore, a consensus was reached regarding the implementation of a safety plan, which involved the designation of two of the psychiatrists as contact persons in the event of a psychiatric deterioration among co-researchers with lived experience within the workshops or the project process in general. Additionally, the roles and responsibilities of the Core team and the study advisory group were clearly delineated.

#### 3.1.2 Evaluation of the workshops

**Figure 1:**
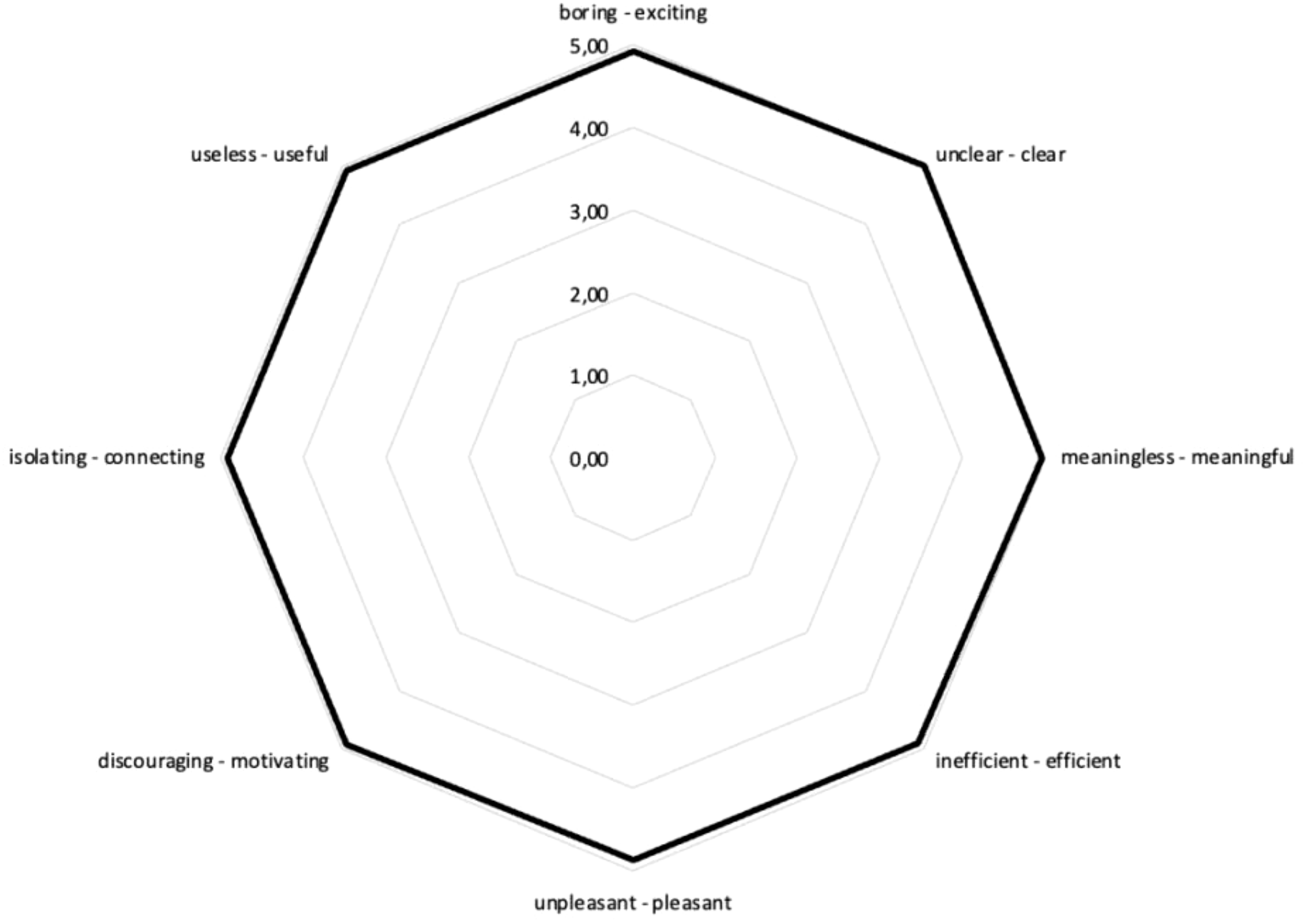
Semantic differential of workshops’ atmosphere. The semantic pairs are mapped on spider web. 1 indicating the negative pole (e.g., boring) and 5 the positive pole (e.g., exciting). The black line indicates mean ratings of four co-creative workshops.

A descriptive analysis of each workshop was performed, as shown in **Table 1**. The evaluation of the workshops showed that overall, the participants were very satisfied with the workshops. Participants rated the atmosphere in the workshop, satisfaction with the workshop, involvement in the workshops, takeaways for their daily routine, and aspects of co-creation covered in the workshop as highly satisfying (**Figure 1**). Participants also experienced the workshop atmosphere as exciting, clear, meaningful, efficient, enjoyable, motivating, connecting, and useful, as shown in **Figure 1**. The results indicate that researchers and co-researchers were actively involved in the process and that their voices were heard. Furthermore, researchers and co-researchers established an effective and enjoyable collaboration between them, which also led to further collaboration in a follow-up project.

**Table 1:** Descriptive analysis of workshops’ evaluation.

Dimensions	Workshops									
	Orientation 1 (n=11)		Diagnosis 2 (n=12)		Terminology 3 (n=9)		Treatment 4 (n=11)		Total	
	*M*	*SD*	*M*	*SD*	*M*	*SD*	*M*	*SD*	*M*	*SD*
*Atmosphere in the workshop*	*4,90*	*0,28*	*4,99*	*0,04*	*4,85*	*0,40*	*4,99*	*0,04*	*4,93*	*0,18*
*Satisfaction with workshop*	*4,89*	*0,28*	*4,94*	*0,18*	*4,98*	*0,07*	*4,98*	*0,06*	*4,95*	*0,11*
Selection of participants	4,82	0,40	4,82	0,60	4,89	0,33	5,00	0,00	4,88	0,25
Organization before the event	4,91	0,30	5,00	0,00	5,00	0,00	5,00	0,00	4,98	0,15
Organization and room onsite	4,82	0,40	5,00	0,00	5,00	0,00	5,00	0,00	4,96	0,20
Comprehensibility of language	4,91	0,30	5,00	0,00	5,00	0,00	5,00	0,00	4,98	0,15
Atmosphere/mood in the group	5,00	0,00	4,90	0,32	5,00	0,00	4,91	0,30	4,95	0,18
*Involvement in the workshops*	*4,91*	*0,23*	*4,94*	*0,35*	*4,87*	*0,30*	*4,91*	*0,22*	*4,91*	*0,06*
The participants were easy to follow.	4,91	0,30	4,91	0,32	5,00	0,00	5,00	0,00	4,96	0,18
I was able to influence the content and results of the event.	5,00	0,00	4,83	0,63	4,89	0,33	4,73	0,65	4,86	0,31
I was able to influence the procedure and design of the event.	4,73	0,47	5,00	0,84	4,56	1,01	4,73	0,65	4,76	0,23
Whenever I voiced an opinion, I was taken seriously.	5,00	0,00	5,00	0,00	5,00	0,00	5,00	0,00	5,00	0,00
The atmosphere allowed for raising objections and voicing opposing opinions	5,00	0,00	5,00	0,00	5,00	0,00	5,00	0,00	5,00	0,00
My expectations towards the event were met.	4,82	0,60	4,90	0,32	4,78	0,44	5,00	0,00	4,88	0,25
*Takeaways fom the workshop*	*4,88*	*0,35*	*4,73*	*0,52*	*4,78*	*0,50*	*4,97*	*0,11*	*4,84*	*0,19*
interesting contacts	4,82	0,00	4,60	0,70	4,56	0,73	5,00	0,00	4,75	0,41
knowledge	5,00	0,40	4,80	0,42	4,78	0,44	4,90	0,32	4,87	0,05
ideas and inspiration	4,82	0,65	4,78	0,44	5,00	0,33	5,00	0,00	4,90	0,27
*Overall satisfaction*	*5,00*	*0,00*	*5,00*	*0,00*	*5,00*	*0,00*	*5,00*	*0,00*	*5,00*	*0,00*
*Aspects of co-creation*			*4,57*	*0,72*	*4,63*	*0,58*	*4,87*	*0,38*	*4,69*	*0,17*
The work in the co-creation workshop was solution-oriented.			5,00	0,00	5,00	0,00	5,00	0,00	5,00	0,00
The co-creation workshop was well facilitated.			5,00	0,00	5,00	0,00	5,00	0,00	5,00	0,00
Participation in the co-creation workshop helped me in my work.			4,00	1,70	3,89	1,69	4,64	1,21	4,18	0,28
I enjoyed participating in the co-creation workshop.			4,80	0,42	5,00	0,00	5,00	0,00	4,93	0,24
I will continue to use the results of the co-creation workshop.			4,60	0,84	4,44	0,88	4,73	0,65	4,59	0,12
I will refer others to the co-creation workshop.			4,00	1,33	4,44	0,88	4,82	0,40	4,42	0,47

*M*, mean; *SD*, standard diviation.

### 3.2 Project outcomes

#### 3.2.1 Consensus on project outcomes

During the workshops, the project team co-creatively developed objectives, identified unmet needs and prioritized the outcomes to be executed. The consensually developed project outcomes based on unmet needs were (i) to create greater awareness on psychosis high risk states in the public, (ii) to enhance access to knowledge on UHR specifics and, consequently, (iii) to help destigmatize and facilitate access to specific care services. It was also deemed important to create new perspectives, to enhance collaboration between patients and clinical staff, and to broaden the scope of vision. Methods of execution and assignment of tasks were determined by the team. The project outcomes were realized entirely through co-creative means.

#### 3.2.2 Identification and implementation of unmet needs

##### 3.2.2.1 Jour fixe

In order to provide a regular forum for exchange and contact among individuals with lived experience of UHR, a “Jour fixe” was organized and established by the co-researchers. The framework for the jour fixe meetings was developed during the workshops.

##### 3.2.2.2 Website

In addition, the VOICE research team co-created a website that provides clinical information on UHR states, background on participatory research, and personal reflections on the participatory project process (https://dasvoiceprojekt.at).

A logo was designed by the co-researchers and implemented with the assistance of graphic designers to establish a distinct identity for the project.

##### 3.2.2.3 Social media

As the symptoms of UHR are experienced by adolescents and young adults, social media, i.e., Instagram ^®^, was agreed upon as a suitable format to address a larger number of people in the age group concerned. The Instagram account @theofficialvoiceproject provides information about symptoms that might occur in psychosis risk for those affected or interested in the topic. It also presents coping skills, resilience methods and other topics on mental health awareness, and is operated, designed and managed by a co-researcher with lived experience (https://www.instagram.com/theofficialvoiceproject/).

##### 3.2.2.4 “How to” guide

As part of the participatory research process, a guide was co-created to provide instructions to set up participatory research projects with individuals with lived experience of UHR. Since, to our knowledge, no such guide has been published before, we focused on specific recommendations and checklists for setting up participatory research projects with individuals with UHR experience as co-researchers. The guide has been made freely available on the open data repository Zenodo (36).

##### 3.2.2.5 Dissemination of findings and the project process

Results and outcomes of this project, and respectively the participatory project itself, were presented by members of the Core Team - always represented by a psychiatrist and a co-researcher with lived experience - at scientific and non-scientific conferences to increase awareness and knowledge of UHR states and participatory research.

##### 3.2.2.6 Publications

One of the objectives determined in the initial workshop was for the participants to reflect on the project collaboration itself. As the workshop sessions approached their end, all project collaborators were encouraged by the Core Team to write a short text laying out their views on the course of the project, considering that for everybody concerned, this type of work had been a novel experience. No constraints or instructions were set for the subjects or themes that should be treated in these “reflections” as they were referred to. The idea was to simply give room to any thoughts or remarks on the project that might have occurred during its course. Out of 12 participants, 8 produced such a text on their own time outside of the workshops; the outcomes were discussed together during the last workshop. A recurrence of certain themes touched upon in these reflections could be observed. To further explore the experiences within the participatory research process, a qualitative analysis of the open reflection reports written by project participants was conducted (37).

##### 3.2.2.7 Follow-up project VOICE+

Following up on the established outcomes and identified unmet needs during the VOICE project, funding was acquired co-creatively for a subsequent participatory project, named VOICE + “How to tell”. The aim of VOICE+ was to foster further awareness and knowledge on symptoms and signs as well as coping strategies in UHR states. Since a lack of access to specific information on psychosis high risk states was identified within the VOICE project, handouts for individuals with UHR experience visiting the early psychosis outpatient clinic were co-created providing information on psychosis high risk states for those affected and their care-givers. Further, a psychoeducation manual for professionals is being created to provide general information concerning psychosis high risk states. A public event comprising a graffiti mural adorned with the VOICE logo and QR codes linked to the project website was conducted to raise awareness on UHR states in the public (https://www.instagram.com/theofficialvoiceproject/reels/). Moreover, in a co-creative process, several short videos were produced to depict possible symptoms associated with psychosis high risk states through the perspective of a person with lived UHR experience. Other videos provide a brief theoretical background on attenuated psychotic symptoms and intervention options. Additionally, coping strategies for attenuated psychotic symptoms were presented based on the perspective of those affected.

Further project objectives for future participatory research collaborations included the establishment of event series at educational institutions or events with a specific focus, such as concerts or theatrical performances, the formulation of political demands within healthcare, creation of a mental health podcast, the implementation of a mentorship programm on psychosis high risk states and an interest group representing those affected and their families, as well as the formation of a non-governmental or non-profit organisation for continuation of the project.

### 3.3 Reflection on the psychosis high risk concept/terminology/diagnostic and treatment options

In the workshops, the heterogenous perceptions of the UHR concept and the terminology used in the field of mental health were discussed. This led to a valuable exchange of ideas and insights on all sides, illuminating aspects mentioned by the co-researchers that psychiatrists may not have previously considered. Furthermore, it facilitated a better understanding of medical concepts and their origins and possibilities of research among co-researchers with lived experience. Despite differing perceptions concerning concepts and terms used within the field of mental health, the necessity of an adequate vocabulary and terminology for psychiatric symptoms and states was deemed crucial by both medical professionals and co-researchers with lived UHR experience. Being able to “name a condition” was considered important for facilitating a better understanding between psychiatrists and those affected and reducing a potentially associated taboo.

During the workshops, the most frequently used, commonly recognized terms relevant to the discourse were discussed. This discourse contributed to a deeper understanding and insight on mental health in general and psychosis high risk states in particular from the perspective of those with lived UHR experience: Some co-researcher described terms such as “recovery” and “mental illness” as not particularly helpful, because they were perceived as not entirely acknowledging the concept of a continuum between health and illness. A general desire for graduations instead of a dichotomous view of being either ill or being healthy was evident within the group of co-researchers. The term “diagnosis” was perceived as beneficial by some co-researchers, as it enables the identification of a condition and, consequently, the formulation of coping strategies, help and solutions. Conversely, the term “psychosis” was associated with ideas of an “unendurable and unimaginable catastrophe”, a “complete loss of control” and the “dissolution” of one’s self” by some co-researchers with lived experience, while others considered it to be outdated and unspecific. However, it was not always straightforward to differentiate between the term itself and the symptom/condition in terms of their perceived unendurability for those affected.

Perceptions and attributions regarding the term “ultra-high risk for psychosis” (UHR) were heterogeneous within the group: Some co-researchers appreciated several advantages of the UHR concept concerning a sense of community, belonging, and a feeling of relief as a consequence of a clear description of a state making their experiences more tangible. However, other co-researchers associated the identification of being at increased risk for psychosis with feelings of uncertainty because of a “lack of diagnosis”, or with concern and fear of possible deterioration and a subsequent need for protection. With respect of terminology, some co-researchers with lived experience perceived terms such as “mental illness” and “ultra-high risk for psychosis” as very distressing and almost intolerable. While the term “ultra” within “ultra-high risk for psychosis” was experienced as an imminent threat by some, others viewed it as a wake-up call prompting them to take action on their mental well-being. The terms and the concept of “decline in functioning” or “reduction in functional level”, as part of the high-risk concept, were experienced as economically characterized terms associated with an increasingly meritocratic society by some co-researchers. In general, more ubiquitous terms such as depression were deemed to be perceived as having less negative association, possibly due to a higher social acceptance.

With regard to interventions, the availability of a range of different options, including pharmacological and non-pharmacological treatments, and the possibility of being able to choose between different interventions, was considered helpful. Psychoeducation was associated with a sense of self-efficacy and control, a reduction in fear, and a subsequent reduction in distress. The perspectives of those with lived UHR experience on the psychosis high risk concept, terminology, diagnostic and treatment options gave a deeper understanding and insight into the partly heterogenous perception within this mental health area. This illustrates the need for attention to individual needs and to use a variety of terminology and explanatory models in the field of mental health. Indeed, the use of an adequate terminology for psychiatric conditions was deemed crucial by both medical professionals and co-researchers with lived UHR experience for facilitating a better understanding between psychiatrists and those affected.

### 3.4 Insights from the “how to” guide

The “How to”- Guide on participatory research with individuals with lived experience of Ultra-High Risk for Psychosis (UHR) derived from our experiences and insights from the VOICE participatory research project. It aims to support researchers and co-researchers with lived UHR experience for setting up a participatory research project. It guides through each step of a participatory research project, provides principles, checklists and specific recommendations concerning the involvement of co-researchers with UHR-experience in research (36).

An overview of best practices on setting up a participatory research project with co-researchers with lived UHR experience derived from the “How to” Guide (36) is shown here:

Before the start:

- Plan project budgeting and potential funding before the project starts. Calculate remuneration and financial compensation for the co-researchers with lived experience. The amount of payment should be appropriate to the level of involvement and should not be tokenistic. Besides adequate remuneration for co-researchers with lived experience, calculate budget for meeting venues, travel and conference costs, external supervision and publication fees.
- Start recruitment of co-researchers from the conceptual stage of the project. Include a co-researcher with lived experience already when writing the project proposal/funding proposal.
- Recruit a Core Team and a Study Advisory Group according to defined in- and exclusion criteria. A balanced Core Team/Study Advisory Group should include the same number of co-researchers with lived experience and professionals/clinicians.
- Be aware of diversity when recruiting (e.g., age, background, experience, gender etc.).
- Informed consent and a confidentiality declaration has to be given in written form by all research participants.

During the project:

- Develop a clear job/role description with clarification of the roles of all participants of the Core Team and of the Study Advisory Group. But: Avoid restricting co-researchers with lived UHR experience to one specific task. Be flexible for adapting existing plans.
- Project-related decisions should be decided consensually.
- Determine rules for interaction and communication within the research team and conclude on “No Go’s” during the participatory process.
- When problems arise during the co-creation process, these issues should be documented and addressed within Core Team meetings.
- Provide learning activities for the research team during the project process; tailor the learning activities according to the project aims and to the interest of the co-investigators.
- A safety plan should be developed in case of any psychiatric deterioration or acute crisis of co-researchers with lived experience during the project (e.g., during workshops). A clinician (e.g., psychiatrist) with expertise in UHR states and psychosis should be included in participatory research projects involving individuals with lived UHR-experience.
- Develop a time table for your project activities. Agree on an action plan for activities: *who* is doing *what* until *when*.
- Schedule regular meetings according to the project activities and assign a PPIE – experienced supervisor for project activities, e.g., workshops.

At the end of the project:

- Disseminate research outcomes (e.g., in publications) and present the project together (e.g., at conferences) with co-researchers with lived experience.
- Ensure that co-researchers are informed about project outcomes and publications.
- Mark the ending of a project with a final meeting) and reflect on the learnings, objectives and the project process of the collaboration.

## 4 Discussion

This paper illustrates the participatory process of a co-creative project with collaboration between individuals with lived experience of psychosis high risk states and psychiatrists experienced within this field. Unmet needs of co-researchers with lived UHR-experience included provision of and free access to specific information and knowledge on psychosis risk states, e.g., on social media, a website; opportunities for personal meetings and personal exchange within the group of those affected (“jour fixe”), and the creation of more public awareness and knowledge about UHR in the professional field, e.g., in care institutions. In order to raise awareness, provide free access to information for those affected and stimulate the scientific discourse in the psychosis high risk research community, it was aimed to make the majority of the project results freely available, e.g., via open repositories and open access publications. Another key outcome was gaining valuable subjective insights into the participatory research process with co-researchers at high risk for psychosis.

In general, the body of quantitative research in the field of participatory research and especially evidence for the impact of involvement on the analysis of quantitative data is scarce (38). In line with other, mostly qualitative, studies (39, 40), a positive impact was found for project participants with high levels of overall satisfaction including enjoyment, involvement as well as personal takeaways such as interesting contacts, knowledge and new ideas. Participants felt taken seriously, able to voice opposing opinions and influence content, results, procedure and design of the project process in the workshops. In previous research (41–44), involvement of co-researchers with lived experience was discussed to disclose more sensitive information, a willingness to freely and honestly share opinions and experiences, and consequently, improve data collection due to a potentially adopted terminology and more sensitive approach. In line with other studies reporting benefits from working with peers, i.e., new friendships and opportunity for exchange and support (40, 45–47), project participants reported high levels of connection within the research group and satisfaction with the selection of project participants as well as making interesting contacts. Project participation was experienced as useful, meaningful and motivating in our workshops. Similarly, participants of previous participatory research projects reported involvement as an “valuable stone to work” (39), “meaningful” (48), “encouraging to speak up and out on issues they felt strongly about, advocating for themselves” (45), increasing self-confidence and self-esteem (48–51).

One possible explanation for the positive perception of the participatory research process by the project participants might be the involvement of co-researchers with lived experience from the project’s very beginning. Research found an early involvement into the research process helpful for reshaping and clarification of research questions as well as challenging of persisting assumptions and aims (46, 52, 53).

In a recent meta-analysis, a positive effect of participatory research was found concerning health service access, self-efficacy and physical health. While initially no significant effect was found for individual mental health outcomes, effects were altered to significantly higher for mental health when patients and the public were involved in more than one research level (54). In this project, participants were free to be involved in various research levels and to undertake one or multiple roles and tasks. For example, a co-researcher with lived experience could be part of the core team in a leading and decision-making role and also be in charge of the social media content or present the project and its outcome at a conference. Strength-based roles of co-researchers were created through identification of individual skills that co-researchers brought to the project team, e.g., production of social media content, videography, writing or artistic talents such as graffiti arts. Consequently, the project had a huge benefit by recognizing co-researchers’ expertise, interests and talents resulting in tailor-made co-created tasks in the implementation of project outcomes. While mental or physical health of participants was not assessed within this project, participants reported a high level of satisfaction within the workshops and aspects of co-creation. Future research might address any effect on mental health within different levels of participation in research.

Previous research showed that flexibility to modify and interchange roles fostered versatility and adaptability among co-researchers engaged in participatory research initiatives. This, in turn, diminishes the probability of being restricted to a singular task (55). Within our project process, we reviewed and debated roles and participatory research activities during and between workshops to permit each project participant the chance to change their level of participation and capacity.

It was tried to avoid power imbalances in the project process by consensually agreeing on rules for collaboration, including a right for giving a “veto” and having an equal number of clinicians and experts by experience in the Core Team as well as in the study advisory group. Emotional work, creating a safe space to co-produce and transparency in decision making were further proceedings designed to establish a secure and comfortable environment for all project participants. While quantitative data analysis implicates an atmosphere of excitement, connection and feeling of purpose, our sample size was very small and thus, results cannot be generalized to participatory research in general. Critical voices concerning certain aspects of participation in mental health research, such as unequal relationships and academic privileges, as well as emotional distress and the complexity of a dual identity has to be taken seriously and evaluated in future research (56, 57).

Involvement of individuals with lived experience into the development of research materials was found to improve understandability in patient information sheets in several participatory research projects (39, 43, 44, 58). Future research has to prove in how far our project outcomes including informational handouts for those affected show differences and potential benefits compared to information material produced without any involvement of experts with lived experience.

The relevance and methodological strength of this participatory research project lies in its flexible, fluid, and iterative approach and its co-creative process from the very beginning by already collaborating with a co-researcher with lived experience during the composition of the project application. Participatory research and co-creation were implemented throughout all project activities. Project outcomes were executed in a co-creative manner at all levels.

In participatory research projects with people with lived experience of mental illness (PWLE), the potential dual identity of co-researchers can result in “colliding worlds” (59, 60): While co-researchers offer expertise and firsthand knowledge based on their experiences, their dual identity may make them susceptible to being viewed primarily as patients rather than equal research participants. Achieving a balance between acknowledging their experiences and knowledge as expertise and avoiding stigmatization and power imbalances requires careful consideration. To ensure adequate knowledge among co-researchers on psychosis high-risk states, we conducted brief presentations on specific topics such as assessment and treatment recommendations in psychosis high risk states.

Tokenism can be a potential hazard in participatory research, e.g., when participation with co-researchers is a compulsory condition for funding applications (8). By excluding the co-researchers’ meaningful influence in the project, PPIE can become a “box-ticking” exercise (61) potentially resulting in disempowerment of participants with lived experience (62). To address the issue of tokenism, it is crucial to prioritize authentic engagement, respect, and reciprocity in participatory research. This involves building trusting relationships, valuing diverse perspectives, actively involving participants in decision-making processes, and ensuring that their contributions are acknowledged and incorporated into the research findings and outcomes. By already including a co-researcher during the conceptual stage of the project, we aimed for a non-hierarchical co-creation process from the very beginning. Creating a balanced guidance structure of co-researchers with lived experience and psychiatrists within the core team and the study advisory group with clear role descriptions was another attempt to avoid tokenism as much as possible. Further, in this PPIE project, workshops were evaluated according to different dimensions such as satisfaction, involvement and aspects of co-creation. The findings indicated a positive collaboration with high levels of satisfaction, involvement and co-creation aspects as well as a meaningful and connecting project process. However, the results of this quantitative data analysis are limited due to the small sample size and the purely descriptive analysis.

Other criticisms of participatory research include alleged reliance on anecdotal research based solely on individual experiences, resulting in an assumed inability to generalize conclusions or outcomes to broader (clinical) populations, and concerns about the representativeness of co-researchers (63). The co-researchers involved in VOICE and the subsequent project VOICE+ were highly motivated, most of them with a high level of socio-occupational functioning. Given that individuals at high risk for psychosis vary widely in education, occupation, distress levels, symptoms and comorbidities, it is unclear whether our projects fully represent this heterogeneity. While concerns about representativeness in participatory research are valid, it is equally important to question whether traditional research – often conducted without lived experience – truly captures the full spectrum of perspectives needed for meaningful mental health research (64).

In general, the level and nature of engagement within a participatory process will vary depending on the discipline and type of study. While some research areas, e.g., qualitative mental health studies, may allow for deep involvement of co-researchers with lived experience during all stages of the research process, other disciplines, such as neuroscience, genetics or randomized controlled trials, may present more challenges in direct participation. However, meaningful involvement remains possible and valuable at different stages including shaping research questions, informing ethical considerations, improving recruitment strategies, refining outcome measures and enhancing dissemination of findings.

## 5 Conclusions

In this participatory project, unmet needs of co-researchers with lived UHR experience could be identified in a co-creative process including free access to information on psychosis risk states, opportunities for personal exchange, and the creation of more public awareness and knowledge about UHR in the professional field. Thus, participatory research with co-researchers with lived experiences in the field of prevention and early intervention in psychiatry, such as psychosis high risk states, may result in an increased awareness towards specific mental health conditions, provision of targeted knowledge and information for those affected and stimulation of the scientific discourse in this area. Perceptions of individuals with lived experience are heterogeneous concerning the psychosis high risk concept and terminology within the field of mental health. This heterogeneity of perception illustrates the necessity of addressing individual needs and utilising diverse terminology and explanatory models. A “one-size-fits-all” approach seems therefore not appropriate. The involvement of individuals with lived experience as “experts by experience” facilitates the generation of valuable insights that can be used to continuously refine work materials and terminology in mental health research and clinical work. This ensures that information is disseminated to the intended audience and that those who require it are able to benefit from it. However, despite different perceptions towards mental health concepts and language, the necessity of an adequate terminology for psychiatric conditions was deemed crucial by both medical professionals and co-researchers with lived experience.

As stated by Rose and Rose (65), in order to truly address epistemic injustice, mental healthcare professionals must move beyond merely “listening to the voices” of mental health service users and instead regard the experiences of those impacted as expertise. Apart from epistemic as well as ethical justifications for participation in psychiatry, future studies have to investigate to what extent the participation of people with lived experience of mental illness (PWLE) can contribute to an actual improvement in awareness, treatment and early detection of psychosis high risk states. In conclusion, to enhance prevention in psychiatry, individuals with lived UHR-experience should and have to be included into future research. Future research could explore tailored approaches for integrating PPIE in different study designs, ensuring that contributions are meaningful while respecting methodological constraints. Recognising these nuances will strengthen the impact and applicability of participatory research across disciplines in psychosis research.

## Data Availability

The original contributions presented in the study are included in the article/supplementary material. Further inquiries can be directed to the corresponding author.
